# The Sesquiterpenes*(E)*-ß-Farnesene and *(E)*-α-Bergamotene Quench Ozone but Fail to Protect the Wild Tobacco *Nicotiana attenuata* from Ozone, UVB, and Drought Stresses

**DOI:** 10.1371/journal.pone.0127296

**Published:** 2015-06-01

**Authors:** Evan C. Palmer-Young, Daniel Veit, Jonathan Gershenzon, Meredith C. Schuman

**Affiliations:** 1 Department of Biochemistry, Max Planck Institute for Chemical Ecology, Jena, Germany; 2 Technical Service, Max Planck Institute for Chemical Ecology, Jena, Germany; 3 Department of Molecular Ecology, Max Planck Institute for Chemical Ecology, Jena, Germany; Rutgers University, UNITED STATES

## Abstract

Among the terpenes, isoprene (C_5_) and monoterpene hydrocarbons (C_10_) have been shown to ameliorate abiotic stress in a number of plant species via two proposed mechanisms: membrane stabilization and direct antioxidant effects. Sesquiterpene hydrocarbons (C_15_) not only share the structural properties thought to lend protective qualities to isoprene and monoterpene hydrocarbons, but also react rapidly with ozone, suggesting that sesquiterpenes may similarly enhance tolerance of abiotic stresses. To test whether sesquiterpenes protect plants against ozone, UVB light, or drought, we used transgenic lines of the wild tobacco *Nicotiana attenuata*. The transgenic plants expressed a maize terpene synthase gene (*ZmTPS10*) which produced a blend of *(E)*-ß-farnesene and *(E)*-α-bergamotene, or a point mutant of the same gene (*ZmTPS10M*) which produced *(E)*-ß-farnesene alone,. *(E)*-ß-farnesene exerted a local, external, and transient ozone-quenching effect in ozone-fumigated chambers, but we found no evidence that enhanced sesquiterpene production by the plant inhibited oxidative damage, or maintained photosynthetic function or plant fitness under acute or chronic stress. Although the sesquiterpenes *(E)*-ß-farnesene and *(E)*-α-bergamotene might confer benefits under intermittent heat stress, which was not tested, any roles in relieving abiotic stress may be secondary to their previously demonstrated functions in biotic interactions.

## Introduction

The isoprenoids, produced through the assembly of branched C_5_ units, are the largest class of plant secondary metabolites, with correspondingly many functions in growth, development and resistance to environmental stresses [1]. Volatile hemiterpenes (C_5_, e.g. isoprene) and monoterpenes (C_10_, e.g. limonene), as well as non-volatile diterpenes (C_20_, e.g. carnosic acid) [2] and tetraterpenes (C_40_, e.g. carotenoids) help plants to tolerate abiotic stress. The role of volatile terpenes in abiotic stress tolerance was first suggested by their enhanced emission under stress conditions. Isoprene emission increases under drought, high light and high temperature [3–5]. Similarly, monoterpene emission increases under heat and drought stress [3,6], and, in *Quercus ilex*, under high-temperature ozone stress [7].

Two mechanisms have been proposed to explain how terpenes could ameliorate abiotic stress [8]: Sharkey and colleagues [9,10] hypothesized that isoprene partitions into cell membranes and enhances membrane stability during transient heat stress, a benefit that might be conferred by alkenes in general. The membrane stability hypothesis was supported by isoprene-mediated increases in membrane order [11]. On the other hand, Vickers and colleagues [12] suggested a general antioxidant mechanism by which harmful reactive oxygen species could be quenched by reaction with unsaturated terpene hydrocarbons. This antioxidant mechanism was supported by the high reactivity of isoprene [13], monoterpenes, and sesquiterpenes with ozone [14].

The theoretical functions of isoprene and monoterpenes in abiotic stress tolerance have been empirically supported by inhibiting and supplementing volatile production. Inhibition of isoprene synthesis using fosmidomycin, an inhibitor of DEOXYXYLULOSE 5-PHOSPHATE REDUCTOISOMERASE (DXR), increased the sensitivity of photosynthesis to heat and light stress in *Phragmites australis* [15]. Similarly, fosmidomycin inhibition of monoterpene synthesis increased ozone-induced damage in *Quercus ilex* [7]. On the other hand, external isoprene supplementation increased thermotolerance of photosynthesis in the non-isoprene emitting *Phaseolus vulgaris* [9] and increased tolerance to singlet oxygen in *Rhamnus alaternus* [16]. External monoterpene supplementation likewise ameliorated the effects of heat stress on photosynthetic rate, chlorophyll fluorescence, and redox status in *Quercus ilex* [7].

While these studies provide considerable evidence for the protective roles of terpenes, there are substantial drawbacks to the manipulation of terpene content by external supplementation and biosynthetic inhibition. External supplementation reverses the concentration gradient resulting from endogenous production such that terpene levels are high outside the leaf and low inside, which may exaggerate headspace effects but miss potential within-leaf effects. Fosmidomycin blocks the entire methylerythritol phosphate (MEP) pathway, which is required for synthesis of tocopherols, chlorophylls, and carotenoids, as well as for isoprene and monoterpenes. Thus, the susceptibility of fosmidomycin-treated plants to stress may reflect decreased synthesis of any of these compounds rather than the absence of isoprene or monoterpenes exclusively.

Manipulation of individual terpene biosynthetic genes has enabled targeted evaluation of the *in planta* functions of specific terpenes. By silencing an isoprene synthase in *Populus x canescens*, Behnke and colleagues increased susceptibility to ozone stress [17] and delayed recovery of photosynthesis after heat stress [18]. Ectopic expression of *Populus* isoprene synthases increased thermotolerance of *Arabidopsis thaliana* [19] and improved thermotolerance and ozone tolerance of *Nicotiana tabacum* [20]. However, the roles of monoterpenes in stress tolerance have not been tested by genetic manipulation, and the roles of sesquiterpenes have not been tested at all.

Sesquiterpenes are exceptionally reactive with atmospheric oxidants, particularly ozone [21]. If this reactivity reflects the ability to prevent oxidative damage by quenching harmful reactive oxygen species within plants or their headspace, then sesquiterpenes might mitigate damage even more effectively than isoprene and monoterpenes. The sesquiterpene (*E*)-β-caryophyllene, for example, is 43 times more reactive with ozone than is the monoterpene limonene [14]. Natural emission patterns further hint that these compounds might ameliorate oxidative stress: sesquiterpene emission, like isoprene and monoterpene emission, increases in response to high light and temperature [22], is robust to mild drought stress [23], and increases after wounding and herbivory [24].

In many cases however, abiotic stress factors could alter emission as a side effect, e.g. by increasing the vapor pressure of already-present compounds via increased temperature, or altering stomatal conductance [25]. Hence, increased sesquiterpene emission under these circumstances is not necessarily adaptive for plants. Furthermore, the main source of reactive oxygen species in plants is the chloroplast [26]. Whereas all terpenes shown to enhance abiotic stress tolerance to date are synthesized in plastids, sesquiterpenes are generally synthesized in the cytosol [27], which could constrain their roles in protection against localized oxidative stress in plastids.

Two sesquiterpenes with high potential to protect against abiotic stress are *(E)*-ß-farnesene and *(E)*-α-bergamotene. *(E)*-ß-Farnesene has a rate constant for reactivity with ozone on the same order of magnitude as *(E)*-ß-caryophyllene [14,28]. Both compounds are induced by multiple stressors in a number of species, suggesting that they might mediate abiotic stress tolerance. For example, in *Pinus taeda*, high light and temperature induce *(E)*-ß-farnesene and *(E)*-α-bergamotene emission [29]. In *Zea mays*, emission is induced by wounding, herbivory, and leaf detachment, which can cause dehydration [24]; in *N*. *attenuata*, *(E)*-α-bergamotene is elicited by wounding, herbivory, or application of methyl jasmonate [30].

We hypothesized that *(E)*-ß-farnesene and *(E)*-α-bergamotene counteract the effects of abiotic stresses via antioxidant or membrane-stabilizing activities. We predicted that external supplementation or *in vivo* synthesis of these sesquiterpenes would mitigate the effects of ozone, drought, and UVB radiation on tissue damage, photosynthesis, oxidative stress markers, and plant fitness. To test for *ex planta* antioxidant activity, we supplemented the ozone-sensitive *N*. *tabacum* cv. BelW3 [31] with external *(E)*-ß-farnesene released into the headspace around the plant. To test for benefits of endogenous sesquiterpene production, two transgenic *N*. *attenuata* lines were used [32]: TPS10, transformed with the *Zea mays* terpene synthase gene *ZmTPS10*, whose main products are *(E)*-ß-farnesene and *(E)*-α-bergamotene [33]; and TPS10M, transformed with a point mutant of the same gene that produces *(E)*-ß-farnesene almost exclusively [33]. We compared *(E)*-ß-farnesene- and *(E)*-α-bergamotene-overemitting transgenics with sesquiterpene-poor wild-type plants under three abiotic stress conditions: ozone, drought, and UVB light.

## Methods

### Plant material

*N*. *tabacum* cv. BelW3 seeds, kindly provided by Jean-Louis Verrier (Tobacco Institute of Bergerac, France) and Ted Woodlief (Oxford Experiment Research Station, Oxford, NC), were surface-sterilized with 2% dicholoroisocyanuric acid and sown on Klasmann plug soil (Klasmann-Deilmann GmbH, Geesten, Germany). After 20 d, seedlings were transferred to 2 L soil-filled pots (Fruhstofer Nullerde, Hawita, Vechta, Germany). Plants were grown in walk-in climate chambers (York, Chicago, IL) at 28°C day/ 20°C night, 75% RH, and 16 h photoperiod under 600 W sodium lamps (350 μmol PAR) (Master Sun-T PIA Agro 400, Philips, Turnhout, Belgium).

An inbred wild-type line of *N*. *attenuata* originating from Utah [34] was transformed with *ZmTPS10*, a multi-product terpene synthase whose major products are *(E)*-ß-farnesene and *(E)*-α-bergamotene, or *ZmTPS10M*, a point mutant that produces *(E)*-ß-farnesene almost exclusively [33]. Transgenes were under the control of a constitutive (35S) promoter. Transformation and screening proceeded as described in Gase *et al*. [35] to select homozygous lines with a single, complete gene insertion and emission of the target volatiles. Complete details of characterization have been published [32]. Plants were germinated and cultivated as previously described [36]. One TPS10 line (A.09.389.6) and two TPS10M lines (A.09.596.1 and A.09.334.6) were selected for experiments, which were conducted with plants of the second transformed generation (T2).

### Ozone fumigation

Ozone fumigation was conducted within a walk-in growth chamber (York, Chicago) (26°C day/ 20°C night, RH 65%, 16 h photoperiod, PAR 350 μmol). Four identical 85 cm x 75 cm x 85 cm (l x w x h) transparent acrylic ozone chambers were used for control and ozone treatments. Ozone chambers received 10 L/min of compressed air with intermittent ozone pulses from an ozone generator (Sorbios, Frankfurt); control chambers received compressed air only. Ozone entered through two steel diffusers mounted at opposite corners of the chamber ceiling. A 12 V fan, suspended 10 cm below the chamber ceiling, ensured that ozone was evenly distributed throughout each chamber (schematic diagram, S1 Fig).

Chamber ozone concentration was monitored using an Environnement O341M (Environnement, Canada) ozone meter. Air was drawn through a perforated 6 mm i.d. Teflon tube suspended within the chamber. Generator readings were taken every 3–6 s and relayed to a computer, which recorded the mean ozone concentration from alternate boxes every 30 readings (3–4 min). After each 30-reading sampling period, a corrective pulse of ozone was delivered to the box that had just been measured. To stabilize ozone concentrations between corrective pulses, a manually determined mini-pulse of 50–200 ms was given to each box before each intermediate reading. Mini-pulse lengths were adjusted to achieve target average ozone levels. Ozone was not detectable in control chambers.

### Ozone fumigation of *(E)*-ß-farnesene-supplemented *N*. *tabacum* cv. BelW3

Sesquiterpenes were supplied externally to 5-week-old BelW3 plants by perfuming with cotton tampons (“Sophie”, size “Super”, Netto GmBH, Maxhütte-Haidhof, Bavaria) impregnated with 2.5 mL of either 2 μg/mL or 200 μg/mL *(E)*-ß-farnesene dissolved in acetonitrile. Control tampons contained acetonitrile only. Immediately before ozone fumigation began, 4 tampons per plant were positioned on 20 cm wooden skewers staked among the leaves, surrounding the plant (S2 Fig, Panel A). Two chambers (one solvent control, one *(E)*-ß-farnesene-supplemented) containing 2 plants each were fumigated for 90 min at 300–500 ppb or until leaf damage started to appear. In the high-dose supplementation experiments, concentrations at the lower end of the range were used, because reaction of *(E)*-ß-farnesene with ozone prevented us from achieving 500 ppb in the terpene-supplemented chamber. Experiments were replicated with the treatment groups switched between the two chambers to control for chamber effects. Parallel fumigations in two identical, ozone-free control chambers verified the absence of effects from (*E*)-β-farnesene supplementation alone. Extraction of tampons at intermediate time points showed that >95% of terpenes were volatilized over the first 60 min (S2 Fig, panel B). Plants were returned to the growth chamber after fumigation and photographed after a 48 h recovery period.

### Ozone fumigation of *N*. *attenuata*

Two replicate chambers containing six 1-month-old elongating *N*. *attenuata* plants (2 each of TPS10, TPS10M, and WT) were fumigated with 300 ppb ozone (ozone treatment) or filtered compressed air (pure air control) for 6 h. To avoid ozone quenching due to excessive humidity [13], plants were not watered for 24 h prior to treatment. After fumigation, plants were returned to the glasshouse or climate chamber (26°C day/ 20°C night, RH 65%, 16 h photoperiod, PAR 350 μmol). Leaf tissue for analyzing moisture content, phytohormones (salicylic acid), rutin, oxidative radical absorbance capacity (ORAC) and lipid peroxidation (malondialdehyde, MDA) was harvested immediately. Photosynthesis was measured the following day. Due to slightly slower growth, line 596.1 was noticeably shorter than bolting WT and 389.6 plants in the experiment for which data are shown. Line 596.1 was therefore excluded from the analysis; in each of numerous replicate experiments, responses of 596.1 were neither visibly nor physiologically distinguishable from those of WT.

### Headspace volatile collection

During ozone fumigations of supplemented and transgenic plants, volatiles were sampled from ozone and control chambers by pulling air at 1 L/min out a hole in the side of the box through ozone scrubbers consisting of 8-ply MnO_2_-coated copper gauze (OBE Corporation, Fredericksburg, TX, USA) enclosed in a Teflon fitting [37], and into glass tubes filled with Poropak Q adsorbent (Sigma-Aldrich, St. Louis, MO). Traps were spiked with 300 ng (+)-cuparene internal standard and eluted with 200 μL dichloromethane.

To determine whether sesquiterpene emission of transgenic plants was robust to ozone fumigation, plant volatiles were measured after 3 h of either ozone (300 ppb) or control (pure air) fumigation. Post-fumigation, plants were immediately moved to the glasshouse (26°C, 65% RH, 16 h photoperiod, PAR 350 μmol) and placed into 15 L acrylic cylinders. A vacuum manifold pulled air at 0.4 L/min through glass tubes filled with Poropak Q for 5 h while filtered air was supplied at 3 L/min through Teflon diffusers. Traps were eluted as described above.

### Sesquiterpene extraction from leaf tissue

Within-leaf sesquiterpenes were quantified in *(E)*-ß-farnesene-supplemented *N*. *tabacum* cv. BelW3 (after 60 min of ozone or control fumigation) and untreated *N*. *attenuata* tps10, TPS10M, and WT (elongated plants). Ground leaf tissue (500 mg) was shaken overnight with 2 mL (n)-pentane and 2 μg *(E)*-ß-caryophyllene internal standard. The extract was filtered through Na_2_SO_4_ and silanized glass wool to remove moisture and particulates, and concentrated to 200 μL at room temperature using a rotary evaporator.

### Gas chromatography-mass spectrometry (GC-MS)

Volatiles and leaf extracts were analyzed on separate GC-MS machines using an Agilent 6890 GC coupled to an Agilent 7683 autosampler and Agilent 7683 quadrupole MS. The injector and inlet were held at 220°C. Sample (1 μL) was injected in splitless mode and passed at 2mL/min through an Agilent 19091S-433 HP5-MS column (0.25 mm i.d. x 30 m l x 0.25 μm film thickness) with helium as carrier gas. Oven temperature was held at 40°C for 3 min, increased at 4°C/min from 40°C to 180°C, then raised at 80°C/min to 250°C and held for 3 min. The transfer line was held at 130°C and the ion source at 150°C. Substances emerging from the GC were ionized by electron impact (EI) with 70 eV. Ions with m/z ratios of 33–350 were monitored in scan mode at a signal frequency of 20 Hz. Compounds were identified by comparison of spectra and retention times with commercial standards, except for *(E)*-α-bergamotene, which was identified by comparison with spectra in the NIST database (US Dept of Commerce, Gaithersburg, MD). *(E)*-α-Bergamotene in the *N*. *attenuata* headspace was previously identified in comparison to an essential oil standard [38]. *(E)*-ß-Farnesene (*m*/*z* 93) and *(E)*-α-bergamotene (*m*/*z* 119 for volatiles, *m/z* 93 for leaf extracts) were quantified relative to the internal standard ((+)-cuparene, *m*/*z* 132; or *(E)*-ß-caryophyllene, *m*/*z* 133 for volatiles, *m/z* 119 for leaf extracts) using the indicated ion traces. Peak areas for volatile emissions were converted into compound masses according to response factors obtained by running dilution series of *(E)*-ß-caryophyllene (Sigma-Aldrich, St. Louis, MO), *(E)*-ß-farnesene (Bedoukian Research, Danbury, CT), and (+)-cuparene (Sigma-Aldrich) to make external standard curves. Because no *(E)*-α-bergamotene standard was available, its response factor was assumed to be equal to that of *(E)*-ß-caryophyllene. Within-leaf sesquiterpene contents were expressed relative to the internal standard *(E)*-ß-caryophyllene, added during the extraction to correct for losses due to leaf matrix effects or volatilization.

### UVB treatment

Rosette-stage 1-month-old plants were subjected to chronic UVB light stress in a growth chamber (26°C day/ 20°C night, RH 65%, 16 h photoperiod, PAR 350 μmol). On one side of the chamber, treated plants in1 L pots were arranged in three rows below two 40W UVB lamps (Philips F40 UVB, Philips Electronics, Andover, MA). Control plants were arranged identically on the other side of the chamber, which was divided in half with a black curtain to shield controls from UV. UV lamps were calibrated using a UVB meter (Solarmeter, Solartech, Harrison Township, MI) to yield 50 μW/cm^2^ (range 48–52) UVB (280–320nm) at pot level. Pots were rotated daily to ensure equal treatment intensity for all replicates. Exposure duration was increased by 1 h/day, from 1 h on the first day of treatment up to 6 h on the 6th day, and held at 6 h/day thereafter, a treatment designed to simulate exposure in *N*. *attenuata*’s native environment [39]. Exposure time was centered around the middle of the chamber light period. Photosynthesis and chlorophyll fluorescence measurements were taken on the 6^th^ and 7^th^ days of UVB treatment. Leaves were harvested for tissue analysis (total phenolics and MDA) on the 8^th^ day. Stalk length and number of seed capsules were recorded after 8 weeks of treatment. Because we expected any sesquiterpene-mediated benefits to accrue from within-leaf terpenes, we used the TPS10M line (A.09.596.1, "TPS10M2") with the highest within-leaf terpene concentrations, and a second TPS10M line (A.09.334.6, "TPS10M1") as a control for non-target effects of genetic transformation.

### Drought treatment

The drought experiment was also conducted using the two TPS10M lines (596.1 and 334.6). Plants were grown in a glasshouse during July and August (26°C day, 20°C night, 65%RH, 16 h photoperiod, PAR 300 μmol). Pots (1 L) were layered with three sizes of expanded clay beads (Lecaton, Weber Saint-Gobain, Denmark) and sand [40]. The bottom layer consisted of 6 mm-diameter beads, the second layer of 4 mm-diameter beads, and the top layer of 2 mm-diameter beads overlain with sand. This easily drained substrate mixture enabled rapid manipulation of plant water status. During the pre-treatment period, pots were watered by hand from above with 100 mL pot^-1^ day^-1^ of 0.3 g/L Flory basis fertilizer (Basisdünger, Euflor, Munich) and 0.6 g/L CaNO_3_. Treatment began late in the rosette stage (49 d old). During the treatment, control plants received 50 mL pot^-1^ day^-1^ of 0.3 g/L Flory basis fertilizer and 0.6 g/L CaNO_3_, whereas drought treatment plants received 10 mL pot^-1^ day^-1^ of a 5x fertilizer solution (1.5 g/L Flory basis fertilizer + 3 g/L CaNO_3_). After 6 d of treatment, half the plants were measured for photosynthetic rate and harvested for total fresh mass and chemical assays. Ion leakage was measured from leaf discs; MDA was measured from homogenates of both aboveground (shoot) and belowground (root) tissues; abscisic acid (ABA) was measured in shoots only. Following this harvest, the differential watering regime was maintained for an additional 7d before stalk length was measured (63 d old). Thereafter, watering of all plants was stopped entirely to accelerate the development of seed capsules. Seed capsules were counted at age 74 d.

### Photosynthesis and chlorophyll fluorescence measurements

Photosynthetic rate and chlorophyll fluorescence were measured from the third or fourth youngest fully expanded leaf between 09:00 and 11:00 AM. For photosynthesis, 2 x 3 cm portions of leaves on-plant were analyzed using a LiCOR 6400 XT (Lincoln, NE, USA) at an illumination of 2000 μmol m^-2^ s^-1^ PAR. Incoming air contained 400 μM CO_2_ supplied at a flow rate of 0.5 mL/s. For drought experiments, an open-topped 1 cm^2^ leaf cuvette was used under ambient glasshouse light conditions due to smaller plant size. Genotypes were alternated over the entire measurement period. Chlorophyll fluorescence (F_v_/F_m_) was determined after at least 15 min dark adaptation using an OS-30p fluorometer (Opti-Sciences, Tyngsboro, MA).

### Ion leakage assay

Ion leakage of 6 mm-diameter leaf disks was expressed as relative conductivity, the percentage of total electrolytes leached over a 3 h incubation period, after Tsarouhas *et al*. [41]: Leakage (%) was calculated as (Leakage conductivity final conductivity^-1^) x 100. Discs were punched from one side of the second youngest fully expanded leaf, midway up the leaf blade. The disc was removed from the punch with forceps and placed abaxial side down in 20 mL doubly deionized water in a 50 mL screw-cap tube. “Leakage conductivity” of the water was recorded after 3h incubation at room temperature. “Final conductivity” was recorded after 30 min incubation at 95°C and subsequent cooling of tubes back to room temperature.

### Quantification of plant fitness correlates

Plant height was measured as the perpendicular distance from the base of the shoot to the highest point on the plant. Seed capsules were counted once a developing capsule’s head was fully fused.

### Tissue harvests for chemical assays

Tissue for chemical analyses came from leaves of equivalent developmental stages in all genotypes. Samples were immediately frozen in liquid nitrogen, ground with a mortar and pestle over liquid nitrogen, and aliquoted into 2 mL microcentrifuge tubes containing 2 steel balls each. The frozen leaf tissue was ground again with the steel balls for 1 min at 20 Hz using a Genogrinder (BT&C Inc., Lebanon, NJ). Tissue was stored at -80°C until analysis.

### Lipid peroxidation—malondialdehyde (MDA) equivalent assay

MDA was quantified 2 d after tissue harvest using the thiobarbituric acid (TBA)-reactive substances assay [15]. Ground leaf tissue (50 mg) was extracted with 1 mL 5% w/v trichloroacetic acid (TCA), shaken for 1 min at 20 Hz, and centrifuged 20 min at 13100 *g* and 4°C. Supernatant (300 μL) was transferred to a 2 mL tube and mixed with 600 μL of 0.5% w/v TBA in 20% w/v TCA. Tube lids were punctured twice with a syringe needle to prevent tube explosion. Tubes were incubated for 30 min in a 95°C water bath, cooled for 5 min in ice water, and centrifuged 5 min at 13100 *g*. Samples were transferred in duplicate to a 96-well microplate and analyzed with a Tecan Third Eye spectrophotometer (Tecan, Austria). Non-specific absorbance (600 nm) was subtracted from TBA-adduct absorbance (532 nm); concentration of TBA adducts was calculated from a standard curve of MDA in 5% TCA.

### Oxidative radical absorbance capacity (ORAC) assay

Oxidative radical absorbance capacity was determined 3 d post-harvest according to Gillespie, Chae, & Ainsworth [42]. Ground tissue (25 mg) was extracted with 1 mL 50% v/v acetone/water, shaken 1 min at 20 Hz, and centrifuged 20 min at 13100 *g* and 4°C. The extract was diluted 20-fold in 75 mM potassium phosphate buffer, pH 7.0. A 150 μL aliquot of 0.04 μM sodium fluorescein was dispensed to each well of a 96-well microplate automatically by a Tecan Third Eye spectrophotometer (Tecan, Austria). Diluted extract (25 μL) was added and the plate was shaken at 37°C for 10 min. After recording the initial fluorescence at 485 nm excitation / 530 nm emission, 25μL of freshly prepared 250 mM 2,2’-azobis-2-methyl-propanimidamidedihydrochloride (AAPH) was dispensed automatically by the Tecan spectrophotometer. Fluorescence was measured every 2 min for 60 min. The readings were summed and divided by the initial reading to obtain an approximate integral of total fluorescence over the incubation. This fluorescence integral, a measure of the sample’s ability to retard fluorescein oxidation, was converted into Trolox equivalents by comparison with readings from wells containing Trolox standards.

### Total phenolic content assay

Total phenolic content was measured using the Folin-Ciocalteu assay as previously described [43]. Ground tissue (20 mg) was extracted with 1.5 mL 95% v/v methanol/water, shaken 1 min at 20 Hz and centrifuged 20 min at 13100 *g* and 4°C. Extract (100 μL) was mixed with 200 μL 10% Folin-Ciocalteu reagent and shaken (2 min) before addition of 800 μL 70 mM Na_2_CO_3._ Samples were incubated 2 h in darkness at RT. After the incubation, 200 μL of sample was transferred in duplicate to wells of a 96-well plate. Absorbance was read at 765 nm using a Tecan Third Eye spectrophotometer (Tecan, Austria). Phenolic content was expressed as gallic acid equivalents based on an external standard curve.

### Rutin quantification

Leaf tissue (100 mg) was extracted overnight at -20°C with 1.5 mL 100% methanol. Doubly deionized water (100 μL) was mixed with 90 μL extract in a GC-MS vial microinsert. Diluted sample (20 μL) was injected onto an Agilent HPLC-1100 with a flow rate of 1 mL/min of a gradient of 0.05% aqueous trifluoroacetic acid (TFA) and acetonitrile (ACN) through a 100 x 4.6 mm Chromolith Performance RP-18e column, 100 x 4.6 mm (Merck; Darmstadt, Germany). Acetonitrile concentration was increased linearly from 10% to 34% over 8 min, followed by a 2 min column wash with 100% ACN and a 2 min re-equilibration to a 9:1 ratio of 0.05% aqueous TFA:ACN. Rutin was identified by spectral comparison with a commercial standard (Sigma-Aldrich, St. Louis, MO) and quantified by absorbance at 330 nm based on an external standard curve.

### Phytohormone quantification

Frozen leaf tissue (100 mg) was extracted overnight at -20°C with 1.5 mL methanol containing the labeled internal standards [^2^H_4_]salicylic acid (SA) and [^2^H_6_]abscisic acid (ABA) (60 ng/sample). Supernatants were evaporated to 250 μL in a rotary evaporator and analyzed using an Agilent 1200 HPLC/MS/MS. Sample (5 μL) was injected and eluted with a flow rate of 800 μL/min through an Agilent Zorbax Eclipse DBC18 1.8 μm column. A gradient of 0.05% formic acid and acetonitrile (ACN) began with 5% ACN from 0–0.5 min and progressed linearly to 100% ACN at 4.5 min followed by washing with 100% ACN from 5–5.5 min and 3 min re-initialization at 5% ACN. SA and ABA were quantified relative to the labeled internal standards using MS/MS monitoring for the ion pairs 140.9/97.0 ([^2^H_4_] SA), 136.8/93.1 (SA), 269/159.2 ([^2^H_6_]ABA), and 263/153.2 (ABA) as in Wang *et al*. [44].

### Statistical analyses

For each experiment (ozone, UVB, and drought), treatment effects and genotype-treatment interactions were tested by two-way ANOVA using SPSS (IBM, Armonk, NY). Phytohormone data were log-transformed and moisture data arcsine-transformed for homoscedasticity. Complete statistical results are documented in S1 Table.

## Results

### Reaction of pure (*E*)-β-farnesene with ozone and its protective effects

**Fig 1:**
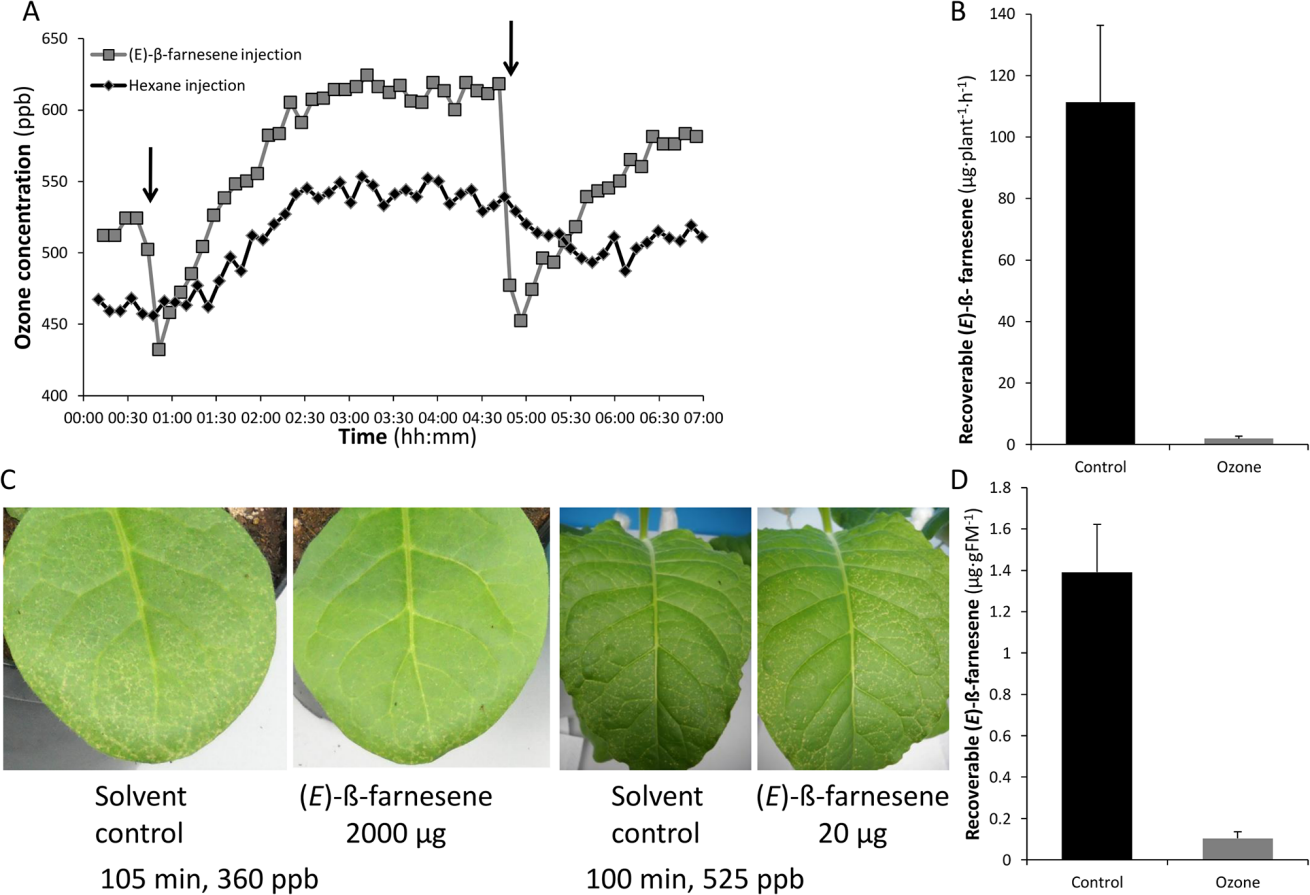
(*E*)-ß-farnesene reacted with ozone to decrease ozone levels. (A) (*E*)-ß-farnesene decreased ozone concentrations in empty chambers. Arrows represent 1 μL injections of either hexane (hexane injection) or (*E*)-ß-farnesene standard ((*E*)-ß-farnesene injection). (B) (*E*)-ß-farnesene disappeared from the chamber headspace during ozone treatment (mean+SEM). Chambers containing two (*E*)-ß-farnesene-perfumed plants each were fumigated with either pure air (control) or 300 ppb ozone (ozone). Air was pulled out of the chamber, through an ozone scrubber, and over Poropak Q adsorbent during the first 100 min of fumigation. (C) Supplementation of the leaf headspace with 2000 μg/plant (*E*)-ß-farnesene, but not with 20 μg/plant, decreased ozone-mediated leaf damage in susceptible *N*. *tabacum* cv. BelW3 plants. (D) Extractable (*E*)-ß-farnesene (mean+SEM) in leaves of supplemented plants after 60 min fumigation with pure air (control) or 350 ppb ozone. FM, fresh mass.

We tested whether the reaction of (*E*)-β-farnesene with ozone, which has been observed in smog chambers [28], could remove substantial amounts of ozone from the air surrounding a living plant, and whether such removal would prevent ozone-induced foliar necrosis. Injecting 1 µl pure (*E*)-β-farnesene into empty chambers containing ca. 500 ppb ozone lowered within-chamber ozone concentrations by >100 ppb within a few minutes (Fig 1A). The disappearance of (*E*)-β-farnesene from chambers containing 350 ppb ozone (Fig 1B) provided additional evidence that the drop in ozone resulted from reaction of ozone with (*E*)-β-farnesene.

To test whether *(E)-*β-farnesene-mediated ozone destruction in the leaf headspace would protect plants, we supplemented the ozone-susceptible *N*. *tabacum* cv. BelW3 with external (*E*)-β-farnesene diluted in acetonitrile and impregnated into cotton tampons. A dose of 2000 μg plant^-1^ h^-1^ visibly reduced ozone-induced foliar necrosis (Fig 1C), but was unrealistically high given the sesquiterpene emission rates reported from plants [45]. In contrast, a physiologically plausible dose of 20 μg plant^-1^ h^-1^ was not protective (Fig 1C). Leaf extractions showed that even during 350 ppb ozone fumigation, some (*E*)-β-farnesene from the 2000 μg plant^-1^ h^-1^ treatment reached and was retained in the leaf, although the 10-fold greater leaf retention in the control fumigation suggested that most *(E*)-β-farnesene was destroyed in the headspace (Fig 1D).

### Sesquiterpene content and emission from transformed *N*. *attenuata* plants

**Fig 2:**
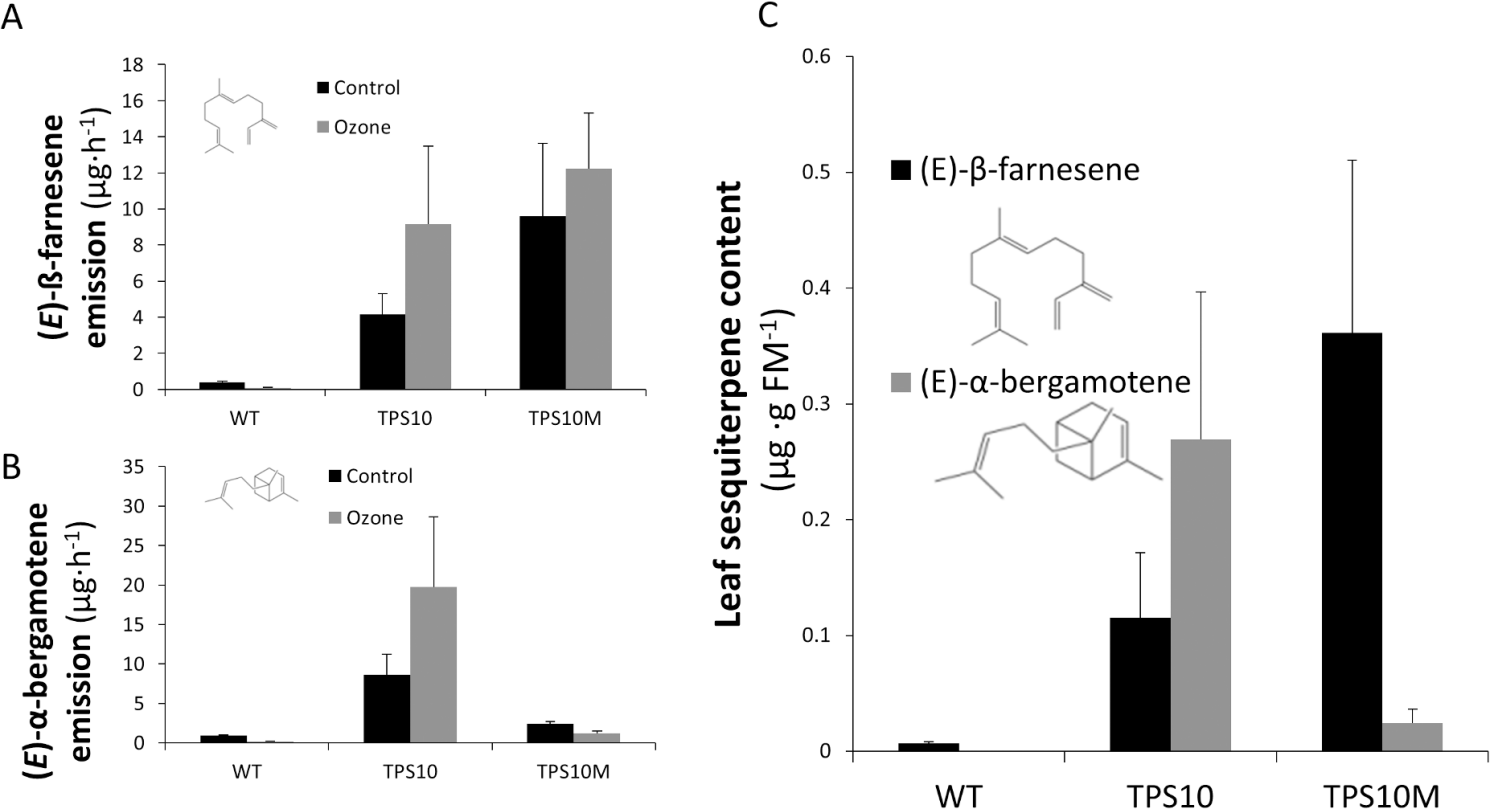
Dominant sesquiterpenes in TPS10 (line 389.6), TPS10M (line 596.1), and WT (wild-type control) *N*. *attenuata*. Retention of (A) (*E*)-ß-farnesene (mean+SEM) and (B) (*E)*-α-bergamotene emission after 3 h ozone fumigation at 300 ppb. Black bars, compressed air control; gray bars, ozone-treated. (C) Extractable sesquiterpenes (mean+SEM) from leaves of transgenic and WT plants. Black bars, (*E*)-ß-farnesene; gray bars, (*E*)-α-bergamotene. FM, fresh mass.

External supplementation indicated that the reaction of (*E*)-β-farnesene with ozone could protect plants by destroying ozone in the headspace around leaves. Yet whether endogenous (*E*)-β-farnesene production would be sufficient to achieve leaf protection, either by acting in the headspace or within the leaf itself, remained unclear. To test for benefits of endogenous production, we transformed *N*. *attenuata* with two versions of a maize sesquiterpene synthase expressed behind a constitutive 35S promoter. The enzyme ZmTPS10 (TPS10 line) yielded nearly equal amounts of (*E*)-β-farnesene and *(E)*-α-bergamotene, whereas the point mutant Zmtps10M produced primarily (*E*)-β-farnesene (Fig 2A and 2B).The amount of transgenic sesquiterpene emission dwarfed native sesquiterpene emission from the wild-type (WT) (Fig 2A and 2B). Emission of α-duprezianine, the only sesquiterpene recorded from unwounded WT plants [38], was less than 5% of total emission in either transformed line and undetectable in leaf extracts. Emission rates were similar in quantity to the lower 20 μg/plant dose used in the BelW3 experiments and robust even to acute ozone exposure (3 h, 300 ppb) (Fig 2A and 2B). TPS10 plants tended to emit higher levels of both sesquiterpenes following ozone exposure, although these increases were not statistically significant (Welch’s t-test, (*E*)-β-farnesene: *t*(2.29) = 1.11, *p* = 0.36; *(E)*-α-bergamotene: *t*(2.35) = 1.21, *p* = 0.33). Emission of the TPS10- and TPS10M-catalyzed sesquiterpenes from the transformed *N*. *attenuata* lines was approximately twice as high as the sesquiterpene emission rate measured from herbivore-damaged maize plants, and exceeded undamaged plant emission by an order of magnitude [22]. Within-leaf sesquiterpenes (Fig 2C) qualitatively resembled emitted sesquiterpenes; within-leaf concentrations averaged 0.4 μg/g fresh mass and were on the same order of magnitude as the 1.4 μg/g observed in BelW3 leaves during the high-dose (2000 μg/plant) external supplementation experiments without ozone treatment (Fig 1D). Leaf sesquiterpene pools in the transformants were similar to the 0.733 μg/g reported by Köllner and colleagues for seedling-stage, herbivore-elicited maize leaves [46], which was the highest value recorded among all tissue types and developmental stages.

### Ozone treatment of sesquiterpene synthase transformants

**Fig 3:**
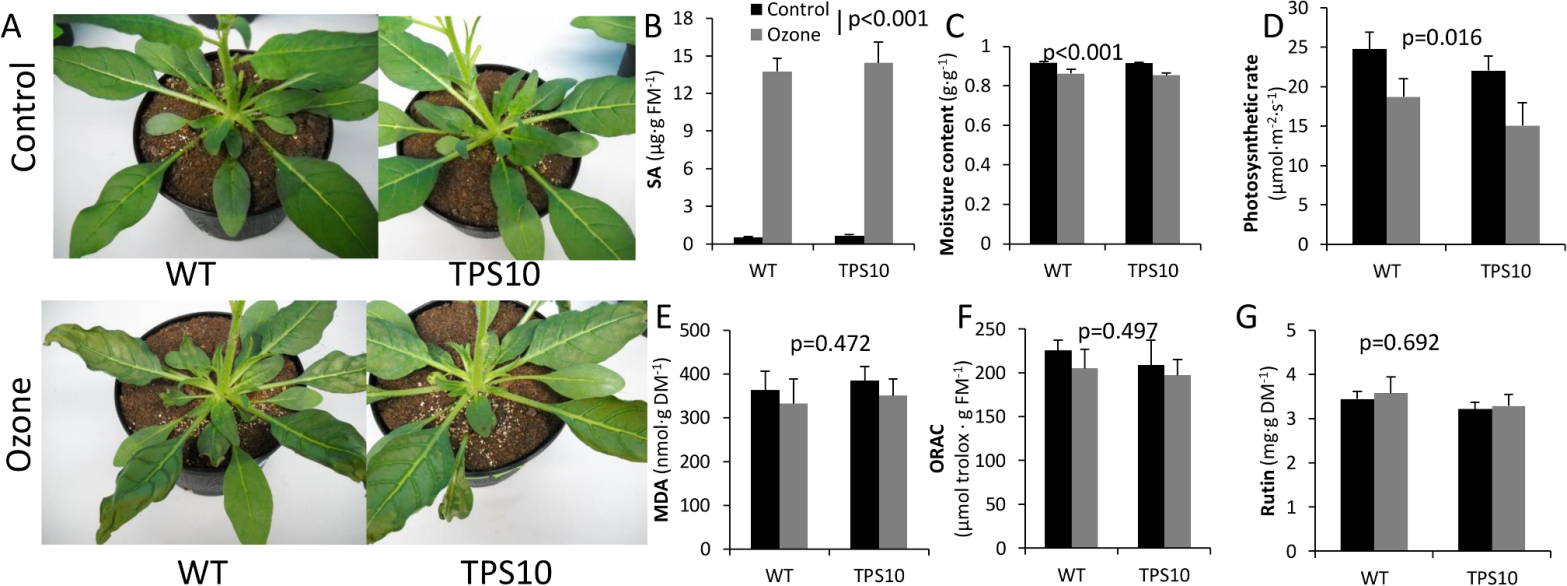
Effects of ozone treatment (6h, 300 ppb) on TPS10 and WT *N*. *attenuata*. Control: black bars; ozone-treated: gray bars. (A) Representative rosettes of ozone-treated and control plants. P-value of ANOVA for overall treatment effect and mean+SEM (n = 4) for (B) salicylic acid (SA) in ozone-treated and control leaves; (C) leaf moisture; (D) photosynthetic rate at 2000 μmol irradiance (E) lipid peroxidation measured in malondialdehyde (MDA) equivalents; (F) Oxidative Radical Absorbance Capacity (ORAC); and (G) rutin, a flavonoid glycoside that was the most abundant phenolic compound in leaf extracts. All measurements were made from leaf tissue harvested immediately post- fumigation except for photosynthesis, which was measured from an on-plant leaf on the following day. DM, dry mass; FM, fresh mass.

Sesquiterpene synthase-transformed and WT *N*. *attenuata* had similar visible and physiological responses to ozone treatment. Leaf necrosis was apparent to equal extents in TPS10 and WT after the 6 h, 300 ppb ozone treatment (Fig 3A). Ozone-fumigated leaves sustained 20-fold increases in salicylic acid (SA, Fig 3B; treatment: *F*(1, 12) = 734.3, *p*<0.001) and significant decreases in moisture content (Fig 3C; treatment: *F*(1, 15) = 25.95, *p*<0.001)) and photosynthetic rate (Fig 3D; treatment: *F*(1, 15) = 7.81, *p*<0.016), all common effects of ozone exposure [47]. A two-way ANOVA showed no significant genotype by treatment interaction in any of these metrics (SA: *F*(1, 12) = 0.88, *p* = 0.37; moisture: *F*(1, 15) = 0.07, *p* = 0.80; photosynthetic rate: *F*(1, 15) = 0.04, *p* = 0.85). Surprisingly, ozone treatment had no significant effect on three markers of oxidative stress in leaves: malondialdehyde (MDA), a product of lipid peroxidation (Fig 3E; treatment: *F*(1, 12) = 0.55, *p* = 0.47); oxidative radical absorbance capacity (ORAC) (Fig 3F; treatment: *F*(1, 12) = 0.49, *p* = 0.50); and rutin (Fig 3G; treatment: *F*(1, 12) = 0.16, *p* = 0.69), a flavonoid glycoside that dominates the leaf phenolic profile of *N*. *attenuata* and whose induction plays a role in UVB tolerance [39]. A TPS10M line (596.1, hereafter "TPS10M2") was also included in the experiment, but results from this line were omitted due to its delayed stem elongation: the leaves of the shorter TPS10M plants were further away from the ozone inlets at the top of the chamber and likely did not experience equivalent ozone concentrations. In numerous replicate experiments, responses of this TPS10M line to ozone did not differ from those of WT (S3 Fig).

### UVB treatment of sesquiterpene synthase transformants

To directly test for within-leaf antioxidant effects of sesquiterpenes, we exposed sesquiterpene synthase-transformed and WT plants to UVB irradiation. Since we had evidence of (*E)*-β-farnesene’s reactivity and protective potential from the ozone experiments with BelW3, and we hypothesized that protection against UVB exposure would accrue from within-leaf terpenes, we used the TPS10M line with the highest leaf (*E)*-β-farnesene content (line 596.1, TPS10M2). To control for effects of transformation unrelated to sesquiterpenes, we included a second, independently transformed TPS10M line (line 334.6, TPS10M1).

**Fig 4:**
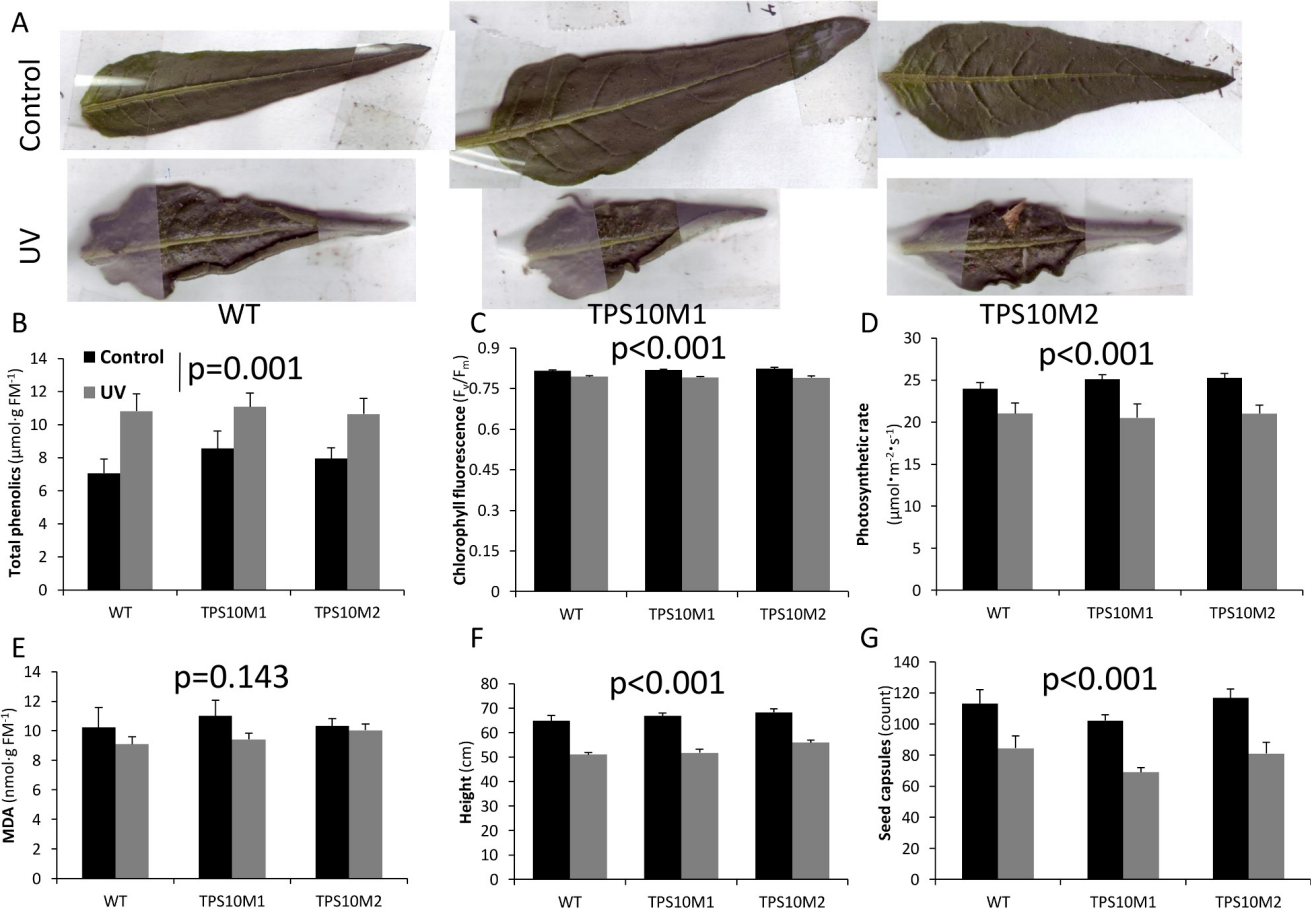
Effects of UVB light treatment on physiology and fitness of TPS10M and WT *Nicotiana attenuata*. Control, black bars; UVB-treated, gray bars. (A) Representative leaves from control and UV-exposed plants. P-value of ANOVA for overall treatment effect and mean+SEM for (B) Total phenolics after 8 d UV treatment, measured in gallic acid equivalents with the Folin-Ciocalteu assay (n = 6); (C) maximum quantum yield of photosystem II, F_v_/F_m_, after 8 d treatment (n = 6);(D) photosynthetic rate at 2000 μmol illumination after 8 d treatment (n = 6); (E) MDA equivalents after 8 d treatment (n = 3; FM, fresh mass); and (F) plant height after 8 weeks’ of treatment (n = 11); (G) total seed capsules after 8 weeks of treatment (n = 11).

The morphological responses of both TPS10M and WT leaves, which assumed a curled appearance and crispy texture, testified to the severity of the UVB exposure (Fig 4A). After 8 days of treatment, phenolic content increased significantly in all lines (Fig 4B; treatment: *F*(1, 28) = 12.73, *p* = 0.001), while chlorophyll fluorescence (Fig 4C; treatment: *F*(1, 29) = 55.20, *p*<0.001) and photosynthetic rate (Fig 4D; treatment: *F*(1, 29) = 24.88, *p*<0.001) were significantly decreased, but no significant differences emerged between responses of TPS10M and WT plants (genotype x treatment, phenolic content: *F*(2, 28) = 0.62, *p* = 0.55; chlorophyll fluorescence: *F*(2, 29) = 0.79, *p* = 0.47; photosynthetic rate: *F*(2, 29) = 0.42, *p* = 0.66). MDA measurements did not show a significant treatment effect (Fig 4E; treatment: *F*(1, 12) = 2.45, *p* = 0.14). The daily UVB exposure was continued for a total of 8 weeks, leading to significant decreases in height (Fig 4F; treatment: *F*(1, 30) = 157.00, *p*<0.001) and seed capsule number (Fig 4G; treatment: *F*(1, 30) = 38.15, *p*<0.001), but again, no differences in responses to treatment appeared between genotypes (genotype x treatment, height: *F*(2, 30) = 0.50, *p* = 0.61; seed capsule number: *F*(2, 30) = 0.16, *p* = 0.86).

### Drought treatment of sesquiterpene synthase transformants

**Fig 5:**
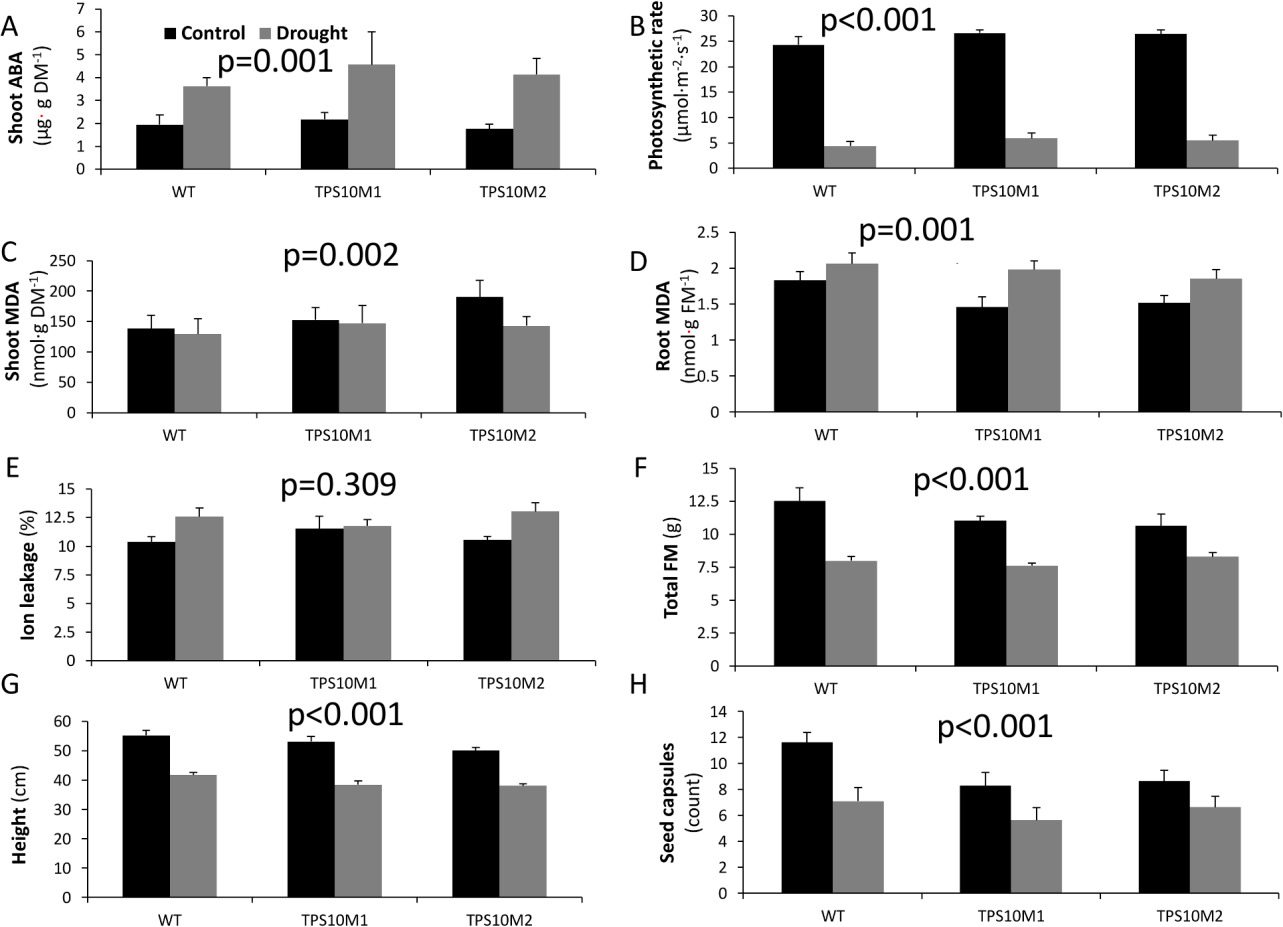
Effects of drought on TPS10M and WT *N*. *attenuata*. Black bars, control; gray bars, drought treatment. P-value of ANOVA for overall treatment effect and mean+SEM (n = 6) after 6 d drought treatment for (A) shoot abscisic acid (ABA); (B) photosynthetic rate; (C) shoot and (D) root malondialdehyde (MDA) equivalents; (E) ion leakage, where leakage is expressed as conductivity of aqueous solution after 3 h room temperature incubation divided by conductivity after boiling 30 min at 95°C; (F) total fresh mass; (G) plant height after 2 weeks’ treatment; (H) total seed capsules at 74 d post-germination, 25 d after treatment began. DM, dry mass; FM, fresh mass.

We tested for (*E*)-β-farnesene-mediated resistance to drought stress using the same two TPS10M lines used in the UV experiment. Within 3 d of treatment onset, all drought-treatment plants raised their leaves, a sign of water stress in *N*. *attenuata*. At the time of tissue harvest, 6 days into the treatment, abscisic acid (ABA) levels were double those in controls (Fig 5A; treatment: *F*(1, 24) = 13.39, *p* = 0.001) and photosynthesis was decreased by 80% (Fig 5B; treatment: *F*(1, 30) = 562.33, *p*<0.001), but responses did not differ between TPS10M and WT (genotype x treatment, ABA: *F*(2, 24) = 0.60, *p* = 0.56; photosynthesis: *F*(2, 30) = 0.12, *p* = 0.89). Although shoot MDA did not show a treatment effect (Fig 5C; treatment: *F*(1, 29) = 1.07, *p* = 0.31), root MDA increased (treatment: *F*(1, 27) = 12.57, *p* = 0.001), but to a similar extent in all lines (Fig 5D; genotype x treatment: *F*(2,27) = 0.71, *p* = 0.50). Ion leakage also increased (treatment: *F*(1, 30) = 8.26, *p* = 0.007). Increases tended to be less dramatic in the (*E*)-β-farnesene emitter TPS10M1 than in WT, but TPS10M2 was no different from WT in its response, nor was the genotype x treatment term significant in an ANOVA (Fig 5E; genotype x treatment: *F*(2,30) = 1.53, *p* = 0.23). Total fresh mass after 6 d of treatment (Fig 5F; treatment: *F*(1, 30) = 50.86, *p*<0.001), plant height after 2 weeks of treatment (Fig 5G; treatment: *F*(1, 60) = 152.19, *p*<0.001), and seed capsule count after an 11 d drying period in both treatment groups (Fig 5H; treatment: *F*(1, 60) = 16.94, *p*<0.001) all showed significant declines with drought, but no differences between the responses of TPS10M and WT (genotype x treatment, fresh mass: *F*(2, 30) = 1.72, *p* = 0.20; height: *F*(2, 60) = 0.53, *p* = 0.59; seed capsule number: *F*(2, 60) = 1.06, *p* = 0.35).

## Discussion

Plant-produced isoprene (C_5_) and monoterpene (C_10_) hydrocarbons have been demonstrated to protect plants from oxidative stress. We tested whether sesquiterpene (C_15_) hydrocarbons, many of which react rapidly with ozone, also confer such protection when externally supplemented or internally generated. After demonstrating the ozone-quenching properties of the sesquiterpene *(E)*-ß-farnesene in empty fumigation chambers (Fig 1A), we showed that external supplementation with large quantities of *(E)*-ß-farnesene protected plants of an ozone-sensitive *N*. *tabacum* cultivar, BelW3, from visible cell death symptoms of ozone exposure (Fig 1C). In contrast, supplementation with physiologically realistic doses of *(E)*-ß-farnesene was not protective. To test for benefits of endogenous sesquiterpene production, we used *N*. *attenuata* transformed with a *Z*. *mays* sesquiterpene synthase gene (35S::*TPS10*) that produced an *(E)*-ß-farnesene and *(E)*-α-bergamotene blend, or a point mutant of the same gene (35S::*TPS10M*) that produced primarily *(E)*-ß-farnesene. Augmenting endogenous sesquiterpene production by engineering these genes into *N*. *attenuata* did not enhance tolerance to oxidative stress under conditions of ozone, UVB, or drought.

### A high dose of external *(E)*-ß-farnesene prevents leaf damage from ozone

Experiments with the ozone-sensitive BelW3 *N*. *tabacum* cultivar demonstrated the potential of *(E)*-ß-farnesene to quench headspace ozone (Fig 1A) and diminish damage, but only when supplied in large amounts (Fig 1B). Although supplementation with 2000 μg plant^-1^ h^-1^ conferred visible benefits (Fig 1C), this sesquiterpene supply rate is two orders of magnitude higher than emission rates from herbivore-damaged *Z*. *mays* [22] or young *Solanum tuberosum* [48]. In contrast, a biologically realistic dose of *(E)*-ß-farnesene (20 μg plant^-1^ h^-1^) was not protective (Fig 1C). Judging from the low, but detectable, amounts of *(E)*-ß-farnesene recovered from supplemented, ozone-fumigated leaves (Fig 1D), ozone levels in the vicinity of the leaf were likely very low; otherwise, ozone would have quickly destroyed any terpenes on the leaf itself.

External supplementation experiments do not mimic the within-leaf sesquiterpene distribution resulting from *in situ* biosynthesis. To clarify the effects of endogenous sesquiterpene production, we used genetic transformation to augment production of two sesquiterpenes, *(E)*-ß-farnesene and *(E)*-α-bergamotene, in the ecological model plant *Nicotiana attenuata*. The transformants grew and developed normally and had no significant physical or chemical differences from WT besides emission of the target volatiles [32]. Sesquiterpene emission was robust to ozone fumigation and even tended to increase in TPS10 plants (Fig 2A). This trend of increased emission, which was also observed in these lines following herbivore elicitation, was unexpected given the expression of TPS10 under the control of a constitutive promoter, and may reflect stress-induced increases in the availability of sesquiterpene precursors [32]. Because sesquiterpene levels in leaves of the transgenics were similar to the amounts of *(E)*-ß-farnesene recovered from leaves of BelW3plants externally supplemented with 2000 μg/plant (Fig 1D), our transgenic lines enabled us to judge whether the protective effects conferred to BelW3 plants from *(E)*-ß-farnesene were mediated by within-leaf activity.

#### Endogenous sesquiterpene formation did not confer ozone tolerance

Given the demonstrated reactivity of sesquiterpenes with ozone, we expected that sesquiterpene production might reduce ozone concentrations in the leaf boundary layer, as suggested for isoprene [13], or provide an *in planta* benefit. Based on the mechanisms proposed for sesquiterpene function, sesquiterpenes can be hypothesized to reduce levels of reactive oxygen species (ROS), especially in lipophilic cellular compartments, and thereby reduce oxidative damage and alter ROS-mediated signaling cascades [12]. Visible lesions and changes in SA level, moisture content, and photosynthetic rate (Fig 3A–3D) showed that our ozone treatment was harsh enough to impact even the stress-resistant desert annual *N*. *attenuata* [34]; shorter or less intense fumigations produced no apparent effects (personal observation). However, the TPS10 line did not differ from WT in its response to treatment (Fig 3), providing no evidence for a protective effect of endogenous sesquiterpene production.

It is possible that the proximity of WT and transgenic plants in the fumigation chambers could have enabled WT to benefit from transgenic neighbors’ emissions. However, our experiments with BelW3 showed that (*E*)-β-farnesene emitted at 20 μg plant^-1^ h^-1^ had a short lifetime in the chamber headspace and did not confer benefits to neighboring plants. Diffusion effects [49] and ozone-mediated destruction create a steep decline of sesquiterpene concentrations with distance from the source. Such a pronounced gradient would have permitted sesquiterpene-emitting transgenics, but not nearby WT, to benefit from terpene-mediated ozone quenching in the leaf boundary layer.

The fumigation experiments indicated that sesquiterpenes are only able to protect against ozone-induced oxidative stress by reacting with atmospheric ozone and preventing direct contact of ozone with the leaf. It is likely that the rate of sesquiterpene emission fromour transgenic plants was insufficient to create this protective headspace when confronted with 300 ppb ozone. Fumigation of 300 ppb for 6 h far exceeds normal atmospheric concentrations of 20–50 ppb [50], and so represents an acute stress. However, short-term exposure to lower concentrations of ozone caused no damage to transformants or WT, such that less extreme treatments could not test the protective function of sesquiterpene production. Under chronic stress imposed by low-level ozone concentrations over prolonged periods of time, benefits of sesquiterpene production might have become apparent, but our ozone fumigation setup required constant monitoring during experiments, which precluded manipulation of ozone concentrations over long periods of time. We tested the hypothesis that sesquiterpene emission could improve tolerance to chronic oxidative stress in the UVB and drought experiments.

### *TPS10M-*transformed *N*. *attenuata* did not differ from WT in responses to UVB exposure

*(E)*-ß-farnesene emission increases in a number of *Pinus* species in response to high light levels [51] and in *Z*. *mays* grown under high light [52], suggesting that this sesquiterpene could confer tolerance to light stress. Plant responses to high light may be adaptive responses to protect against UVB radiation, which in natural environments increases in proportion to photon flux density. However, we found no evidence that *(E)*-ß-farnesene was protective against the effects of UVB. UVB treatment strongly affected leaf morphology (Fig 4A), phenolic content (Fig 4B), chlorophyll fluorescence (Fig 4C), and photosynthetic rate (Fig 4D), but TPS10M and WT lines were similarly affected. UVB exposure damages photosynthetic machinery in the chloroplast, particularly photosystem II [53]. Because sesquiterpene synthases, including ZmTPS10, and their substrate, farnesyl diphosphate, are co-localized in the cytosol [54], sesquiterpene synthesis might not offer protection against local stress in the chloroplast. However, the ability of even externally supplied monoterpenes to improve plastid thermotolerance, as measured by chlorophyll fluorescence [6], suggests high mobility of volatile terpenes within and among cells. Non-polar hydrocarbons, including sesquiterpenes, should diffuse readily across membranes and partition into plastidial and cellular membrane systems, where they could protect against UVB-induced oxidative damage [53,55].

### Drought treatment had similar effects on TPS10M and WT *N*. *attenuata*

If sesquiterpenes can diffuse across membranes and quench reactive oxygen species *in planta*, they should be able to alleviate drought stress, which generates oxidative stress in multiple cellular compartments [56]. Correlations between drought stress and terpene emission suggest that terpenes confer drought stress tolerance. Natural emission of isoprene is typically not reduced by drought [57]. Although monoterpene emission is repressed under severe drought stress [8,58], in wild *Cistus monspeliensis*, total terpene emission was 70-fold higher during hot, dry Mediterranean summers than during winters [59]. In *Citrus sinensis*, sesquiterpene emission is maintained under mild drought stress [23]. In *Z*. *mays*, emission of *(E)*-ß-farnesene is stimulated by herbivory and leaf excision [24], which increase rates of leaf dehydration, as well as by growth under low humidity, a form of mild water stress [52]. In our drought experiment, seven of the eight metrics shown in Fig 5 were significantly affected by drought treatment, demonstrating that plants were under measurable stress. However, (*E)*-β-farnesene conferred no protection against this stress at either the chemical or whole-plant level: the effects of drought on ABA accumulation, electrolyte leakage, photosynthetic rate, and reproductive fitness correlates were similar in *N*. *attenuata* TPS10M and WT plants.

### Potential antioxidant and membrane-stabilizing effects of *(E)*-ß-farnesene and *(E)*-α-bergamotene

Although our results did not demonstrate that sesquiterpenes ameliorate oxidative stress, they also do not directly refute the antioxidant hypothesis for terpene-mediated abiotic stress tolerance [12], since the parameters expected to be most affected by terpenes did not exhibit significant responses to treatment. For example, the antioxidant activity of sesquiterpenes would have been predicted to prevent elevations in MDA and preserve antioxidant capacity. However, ozone treatment did not significantly alter MDA (Fig 3E), oxidative radical absorbance capacity (Fig 3F), or phenolic content (Fig 3G). Shoot MDA also did not change under UVB exposure (Fig 4E) or drought (Fig 5C). The only instance of MDA elevation was seen in roots under drought stress (Fig 5D), but the effects of treatment were the same on TPS10M and WT. A plant that displays more dramatic changes in MDA after stress could offer a direct test of the antioxidant hypothesis as it applies to (*E)*-β-farnesene.

Our results also do not directly challenge the membrane stabilization-mediated thermotolerance hypothesis [10]. Sharkey and colleagues suggested that the beneficial effects of isoprene on membrane stabilization are not realized unless damage occurs within a particular temperature range [10]. Indeed, the effects of isoprene on membrane characteristics were more pronounced at 40°C than at 20°C [60], whereas our experiments were conducted at 26–28°C. If sesquiterpenes are also membrane stabilizers, they could have a similar temperature range in which they are effective, and our experiments may have been conducted outside of this range. Like antioxidant activity, membrane stabilization would be expected to prevent MDA accumulation, but MDA levels were seldom affected by treatment. Membrane stabilization should also have preserved membrane integrity, which we measured as ion leakage, but the drought treatment was the only treatment that significantly increased ion leakage (Fig 5E, data for other treatments not shown). In this case, one TPS10M line seemed to suffer less drought-induced ion leakage than WT, but the second TPS10M line did not differ from WT.

Ours is not the first study to report that terpene supplementation does not increase tolerance to abiotic stress, but our study withstands the criticisms directed at earlier studies that showed a lack of benefit from added isoprene. Loivamäki and colleagues [61] found no enhancement of photosynthetic thermotolerance in isoprene synthase-transformed *A*. *thaliana*, but this conclusion has been questioned [12] because of low emission rates from transformants. Our transgenic lines emitted sesquiterpenes at rates comparable to, or greater than, those found in herbivore-elicited *Z*. *mays* [24]. Isoprene supplementation did not enhance redox status or membrane integrity of isolated *Spinacea olearacea* thylakoids [62] or increase thermotolerance in leaf discs of four isoprene-emitting species [63], but these papers did not test whole-plant responses. In our experiments, we used intact plants producing endogenous sesquiterpenes at the high end of natural levels, but still observed no protective effects.

A subtle protective influence of sesquiterpenes might be undetectable against the stress-adapted background of *N*. *attenuata*. This wild tobacco has weathered extremes of temperature, drought, and light stress over its evolutionary history in the deserts of the American southwest [34] and has likely been selected for many different modes of oxidative stress tolerance besides production of volatile terpenes. For example, antioxidant capacity strongly depends on constitutive and induced glutathione, ascorbate, and tocopherols, as well as the peroxidases and reductases that regenerate their activity [64]. Using more sensitive species, such as *N*. *tabacum*, might facilitate detection of terpene function in abiotic stress resistance. Genetic transformation of *N*. *tabacum* has previously revealed a small but significant role of isoprene production [20] in tolerance to heat stress and ozone, as well as the role of antioxidant enzymes in resistance to oxidative stress from salt and the herbicide paraquat [65], and might likewise elucidate functions of larger terpenes.

### *(E)*-ß-farnesene and *(E)*-α-bergamotene may function in biotic, rather than abiotic interactions in *Nicotiana attenuata*

Our results argue against utility of *(E)*-ß-farnesene and *(E)*-α-bergamotene in resistance to abiotic stress. Previous work suggests that these two sesquiterpenes may instead function against biotic stresses. *ZmTPS10* transformation of *A*. *thaliana* attracted the parasitic wasp, *Cotesia marginiventris*, to its Lepidopteran host [66]. Similarly, *(E)*-α-bergamotene was emitted from *N*. *attenuata* in response to herbivore-mediated jasmonate signaling [67] and attracted natural enemies of *N*. *attenuata*’s herbivores [68]. *ZmTPS10*-produced sesquiterpenes may also primarily mediate biotic interactions in their native maize, where emission is more responsive to herbivory than to light, temperature, and humidity [22]. Bioassays and field experiments with terpene synthase-transformed and—silenced plants could effectively test the utility of *(E)*-ß-farnesene and *(E)*-α-bergamotene in biotic interactions [69].

Another role for sesquiterpenes might be as within- or between-plant signals. The enhanced growth of isoprene-emitting *A*. *thaliana* plants under heat stress [61], and the lack of isoprene-mediated thermoprotection in either isolated membranes [62] or leaf discs [63], would be consistent with the hypothesis that terpenes ameliorate stress by activating within-plant signaling cascades rather than by direct reaction with oxidants. Because of their reactive nature, sesquiterpenes might influence hydrogen peroxide signaling [8] or prime responses to further stress in neighboring cells, organs or individuals in a phenomenon analogous to volatile-mediated induction of herbivore resistance [70]. In *N*. *attenuata*, such signaling seems unlikely, since TPS10 and TPS10M transformants were chemically indistinguishable from WT and did not influence the defense physiology of WT neighbors when the two genotypes were grown together in the same pot [32]. Effects of sesquiterpene perception on other species could be examined with microarrays and metabolomics.

Sesquiterpenes other than *(E)*-ß-farnesene and *(E)*-α-bergamotene, including the highly ozone-reactive α-humulene or (*E*)-β-caryophyllene [14], might be of more benefit against oxidative stress. Our experiments tested only two of the more than 25,000 known plant terpenes [1], a diversity that may reflect specialized roles of each compound or blend. Future experiments employing other compounds and conditions may reveal each compound’s functional niche. Correlations between environmental factors, expression profiles of terpene synthase genes, and emission patterns of the corresponding terpene volatiles provide clues to terpene functions, while alteration of terpene biosynthetic genes enables targeted evaluation of each compound’s phenotypic effects.

## Supporting Information

S1 Fig Schematic diagram of ozone fumigation chambers. (DOCX)

S2 Fig External terpene supplementation experiments with *N*. *tabacum* cv. BelW3. (DOCX)

S3 Fig Effects of ozone treatment on TPS10, TPS10M2, and WT *N*. *attenuata*. (DOCX)

S1 Table Complete ANOVA results for effects of ozone, UVB, and drought treatment on TPS10, TPS10M, and WT *N*. *attenuata*. (XLSX)
