# Supplementary material for: The Sesquiterpenes(E)-ß-Farnesene and (E)-α-Bergamotene Quench Ozone but Fail to Protect the Wild Tobacco Nicotiana attenuata from Ozone, UVB, and Drought Stresses
Source: PLoS One. 2015 Jun 1;10(6):e0127296. doi: 10.1371/journal.pone.0127296 (PMC4452144; doi:10.1371/journal.pone.0127296)
Supplement: S1 Fig — (DOCX) [file pone.0127296.s001.docx]

**S1 Fig. Schematic diagram of ozone fumigation chambers.** Output from an ozone generator was diluted with filtered compressed air and mixed with a fan mounted on the chamber ceiling. Ozone concentration was monitored by sampling air alternately from each chamber through a ring of perforated Teflon tubing ("Ozone sampling ring") connected to an ozone meter. Ozone meter readings were relayed to a computer, which regulated the delivery of air from the ozone generator into each box. Ports in the top of the chamber enabled injections of volatile standards, while a port in the side of the chamber enabled sampling of chamber volatiles.
