# Supplementary material for: The Sesquiterpenes(E)-ß-Farnesene and (E)-α-Bergamotene Quench Ozone but Fail to Protect the Wild Tobacco Nicotiana attenuata from Ozone, UVB, and Drought Stresses
Source: PLoS One. 2015 Jun 1;10(6):e0127296. doi: 10.1371/journal.pone.0127296 (PMC4452144; doi:10.1371/journal.pone.0127296)
Supplement: S2 Fig — (DOCX) [file pone.0127296.s002.docx]

**S2 Fig. External terpene supplementation experiments with *N. tabacum* cv. BelW3.**

**(A)** Setup used for external terpene supplementation experiments with *N. tabacum* cv. BelW3. As described in Materials and Methods, cotton tampons were impregnated with 2.5 mL of either 2 µg/mL or 200 µg/mL *(E)*-ß-farnesene dissolved in acetonitrile. Control tampons contained acetonitrile only. Immediately before ozone fumigation began, 4 tampons per plant were positioned on 20 cm barbecue skewers staked among the leaves, surrounding the plant.

**(B)** Loss of (*E*)-β-farnesene (relative to initial mean + SEM) from tampons used for supplementation experiments. Tampons were impregnated with 5 µg (*E*)-β-farnesene dissolved in 5 mL acetonitrile and extracted with hexane either immediately or after 60 minutes of fumigation at 0 ppb (Control) or 300 ppb (Ozone). (*E*)-β-Farnesene was quantified using the extracted ion chromatogram for m/z 93.0 employing the GC-MS parameters detailed in Materials and Methods.
