# Supplementary material for: The Sesquiterpenes(E)-ß-Farnesene and (E)-α-Bergamotene Quench Ozone but Fail to Protect the Wild Tobacco Nicotiana attenuata from Ozone, UVB, and Drought Stresses
Source: PLoS One. 2015 Jun 1;10(6):e0127296. doi: 10.1371/journal.pone.0127296 (PMC4452144; doi:10.1371/journal.pone.0127296)
Supplement: S3 Fig — (DOCX) [file pone.0127296.s003.docx]

**S3 Fig. Effects of ozone treatment on TPS10, TPS10M2, and WT *N. attenuata.***

**Figure S3A**: TPS10 (line 389.6), TPS10M (line 596.1 (“TPS10M2”)), and WT plants following a 6 h, 300 ppb ozone fumigation. Plants were photographed 24 h after fumigation.

**Figure S3B**: Rosettes of TPS10, TPS10M, and WT plants following a 6 h, 300 ppb ozone fumigation. Plants were photographed 24 h after fumigation.

**Figure S3C**: Salicylic acid (SA) levels in leaves of TPS10, TPS10M2, and WT plants immediately following 6 h ozone fumigation at 300 ppb. P-value is for ANOVA test of overall treatment effect. In control samples, SA was significantly lower in TPS10 plants than in TPS10M2 (p<0.001) and WT (p<0.001), but there were no significant differences between lines in ozone-fumigated samples. Black bars, control. Gray bars, ozone. Error bars show mean + 1 SEM. Analyses were performed on log-transformed data to meet ANOVA assumptions of homogeneity of variance and normal error distribution.

**Figure S3D**: Moisture content in leaves of TPS10, TPS10M2, and WT plants immediately following 6 h ozone fumigation at 300 ppb. P-value is for ANOVA test of overall treatment effect. There was no significant genotype x treatment interaction (p=0.459). Black bars, control. Gray bars, ozone. Error bars show mean + 1 SEM.

**Figure S3E**: Evan’s Blue staining to visualize cell death in leaves of TPS10, TPS10M2, and WT plants following 6 h ozone fumigation at 300 ppb. Immediately after fumigation, the second oldest stem leaf on each plant was excised with scissors and placed in a plastic dish filled with 0.05% (w/v) Evan’s Blue stain. Leaves were incubated overnight on a shaker table at 30 rpm. After incubation, leaves were rinsed 3 times in deionized water. Pigments were removed in a 70°C water bath with a mixture of 1:3:1 (v:v:v) lactic acid: ethanol:glycerol. Leaves were photographed on a light table.
